# A miniaturized ultra-wideband filter with high rejection and selectivity based on dual-notch bands

**DOI:** 10.1371/journal.pone.0306730

**Published:** 2024-08-02

**Authors:** Mingming Gao, Ya He, Jingchang Nan, Zhenzhi Yang, Congying Wang

**Affiliations:** 1 College of Electronics and Information Engineering, Liaoning Technicial University, Huludao, Liaoning Province, China; 2 Liaoning Key Laboratory of Radio Frequency and Big Data for Intelligent Applications, Huludao, Liaoning Province, China; Model Institute of Engineering and Technology, INDIA

## Abstract

A novel compact and highly selective Ultra Wide Band (UWB) filter is proposed using multimode resonator (MMR) technology. To begin with, the filter’s ultra-wideband performance is achieved by coupling a stepped-triangular multimode resonator with input-output feedlines. Furthermore, dual-notch band characteristics are realized at 6.80 GHz and 9.82 GHz, employing asymmetric coupled lines and the split ring resonator (SRR) methods. Eventually, by using a Defected Ground Structure (DGS), the filter’s correct transmission zero is deepened, further enhancing the out-of-band suppression performance at higher frequencies. The measured results are in excellent agreement with the experimental results, and the filter has a passband range of 3.52-11.68 GHz, a center frequency of 7.59 GHz, an insertion loss of just 0.61 dB, and a return loss of more than 18 dB. The transmission zeros have a rejection capability of more than 47 dB attenuation, and the rectangular coefficient of the filter is 1.34, which is outstanding for filtering out the interference signals in the parasitic passband with superior selectivity. The overall structure is compact, and the size is just 0.41λ_*g*_×0.20λ_*g*_. The filter can be used for UWB system filtering and also to avoid interference from some Wireless Local Area Network (WLAN) IEEE 802.11 series and x-band satellite link frequency bands.

## 1 Introduction

The U.S. Federal Communications Commission (FCC) resolved to use ultra-wideband (UWB) frequency bands for commercial use in 2002, and since then, ultra-wideband technology products have ushered in rapid development. UWB filter, as a critical passive component in the UWB wireless communication system, has received critical attention from scholars at home and abroad [1]. Scholars have researched solutions, such as high and low-pass filter cascade technology, hybrid microstrip/coplanar waveguide technology, Z-transform synthesis technology, and multimode resonator technology [2–4]. Conventional ultra-wideband filters mostly use high and low-pass filter cascade techniques [5, 6]. However, this design method requires aconsiderable number of resonators to be covered to achieve the ultra-wideband effect, which is not conducive to the miniaturization of the device and increases the insertion loss in the passband. In order to solve the shortcomings of the traditional ultra-wideband design, the earliest L. Zhu [7] research team proposed the concept of a multimode resonator. It used the designed step impedance resonator (SIR) in the ultra-wideband filter design, which has a miniature size and improves the out-of-band rejection performance.

Reference [8] accomplished the ultra-wideband design by coupling two oppositely placed microstrip lines with a coplanar waveguide in the bottom plane, and three notches are introduced by using the split ring resonator (SRR), complementary folded split ring resonator (CSRR), and folded split ring resonator (FSRR)structures. However, the insertion loss in the passband is high, and the bandwidth range needs to be increased. From the ultra-wideband indoor civil spectrum specification defined by the FCC, it can be seen that the ultra-wideband wireless communication spectrum overlaps with the existing WiMAX signal band of 3.5 GHz, WLAN signal band of 5 GHz, and X-satellite communication signal band of 8 GHz [9]. In order to avoid the interference of these radio signals, the optimal solution is to introduce transmission notches in the frequency response of the UWB filter to realize the trapping in the overlapping bands. Overlapping bands to realize the stopbands characteristics, the notch bands UWB filter has rapidly become a research hotspot. In reference [10], an ultra-wideband filter is realized by introducing a complementary split-ring resonator. However, the insertion loss of this filter is high, and the out-of-band rejection needs to be better. Reference [11] introduces an open-ring resonator into the I/O feeder to realize a trapped wave ultra-wideband filter. Nevertheless, the size needs to be more significant to meet the current trend of miniaturization. In reference [12], dual-notch performance is realized by microstrip-coplanar waveguide structure. However, the return loss in the passband is low because of the introduction of stopbands performance, and the depth of notches needs to be deepened.

Based on the above problems of the filter’s less-than-ideal notches depth, poor out-of-band rejection, improved band selectivity, and size that cannot meet the current miniaturization requirements, this transcript designs an ultra-wideband filter with dual-notch bands characteristics. Primarily, a novel multimode resonator derived from a stepped impedance triangular resonator is integrated into the horizontal transmission line. The coupling between input-output feedlines and the resonator facilitates the realization of a UWB filter spanning 3.52 to 11.68 GHz, exhibiting an insertion loss below 0.61 dB within the passband and a return loss exceeding 18 dB. Secondly, introducing asymmetric coupled lines and coupling between these lines and the multimode resonator creates the first notch at a center frequency of 6.80 GHz. Additionally, a second notch, with a center frequency of 9.82 GHz, is achieved by coupling an open-loop resonator above the input-output feedlines. Finally, a transmission zero with attenuation reaching up to 53 dB is introduced by etching a C-type SRR structure on the backplane, enhancing out-of-band suppression capabilities. Remarkably, the compact dimensions of the filter, measuring only 0.41λ_*g*_×0.20λ_*g*_, contribute to its miniaturized profile. Furthermore, the notces’ depths are sufficiently deep (greater than 26 dB) with a narrow notch range. Both transmission zeros exhibit amazing rejection performance with more than 47 dB attenuation.

## 2 UWB filter theory and analysis

### 2.1 Analysis and design of MMR with stepped triangular resonator

**Fig 1:**
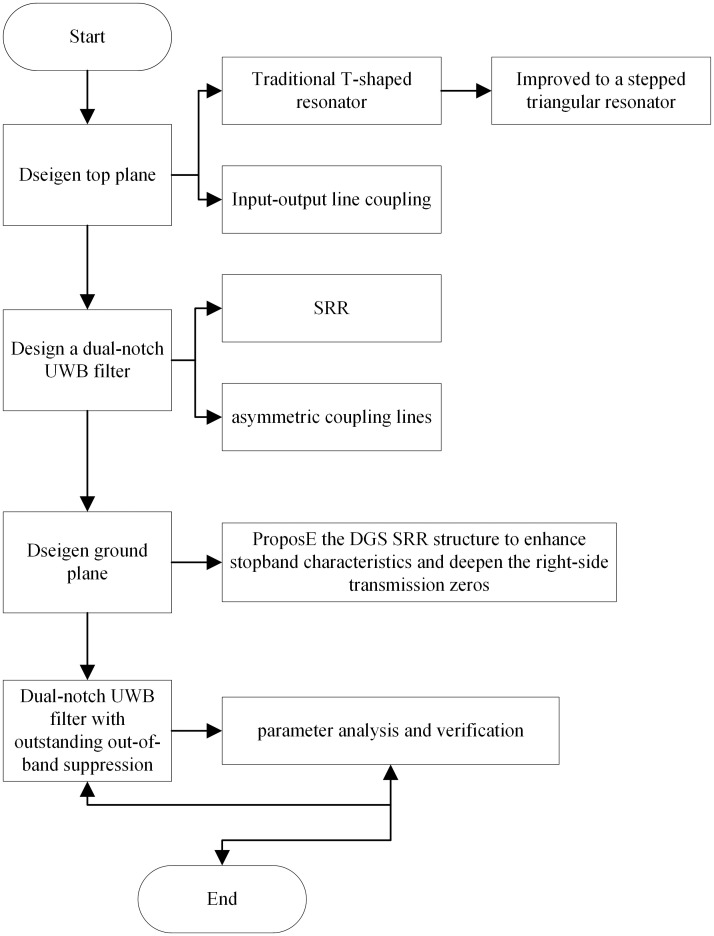
Design flow chart.

**Fig 2:**
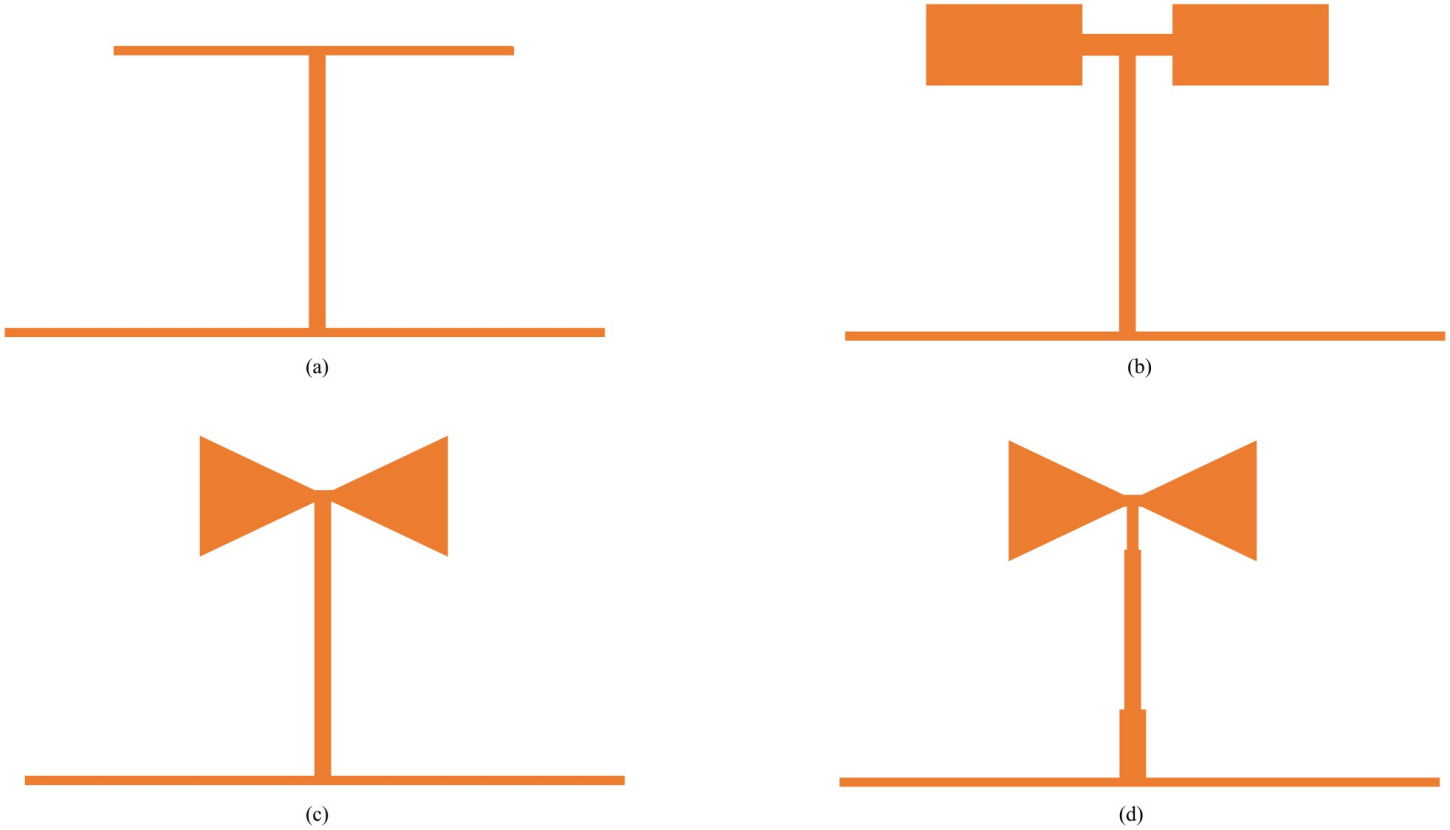
Evolution of multimode resonators. (a) Conventional T-shaped. (b) Half-wavelength shape. (c) Triangular structur. (d) Stepped triangle.

The UWB filter design flow chart in this manuscript is shown in Fig 1. This study proposes a novel multimode resonator based on the theory of multimode resonator, which is obtained by the improved evolution of the traditional T-type resonator. The evolution process is shown in Fig 2, in which Fig 2(a) is a traditional T-shaped resonator; a half-wavelength structure with stepped-impedance resonator branches (SIR) is used to replace the uniform impedance branches (UIR) parallel to the horizontal transmission line of the original traditional T-shaped resonator, as shown in Fig 2(b). The optimization of the high-resistance part of both sides of the SIR branches is transformed into an isosceles triangular structure. Compared with the original rectangular structure, the impedance of the triangular structure is gradual, which can further reduce the effect of the discontinuity of the impedance and the offset, as shown in Fig 2(c); finally, the uniform impedance branch (UIR) perpendicular to the horizontal transmission line is changed to a stepped impedance branch in the triangular resonator as shown in Fig 2(d). Finally, the improved stepped triangular resonator forms a new multimode resonator on the horizontal transmission line.

According to the Transmission line impedance transformation theory, A uniform microstrip line of a certain length can be equated to a resonator whose input impedance can be defined as:

$$\begin{array}{c} Z_{in}=Z_{0}\frac{Z_{L}+jZ_{0}tan\theta }{Z_{0}+jZ_{L}tan\theta } \end{array}$$ (1)

where Z_0_ and Z_*L*_ are the characteristic impedance and load impedance, respectively.

**Fig 3:**
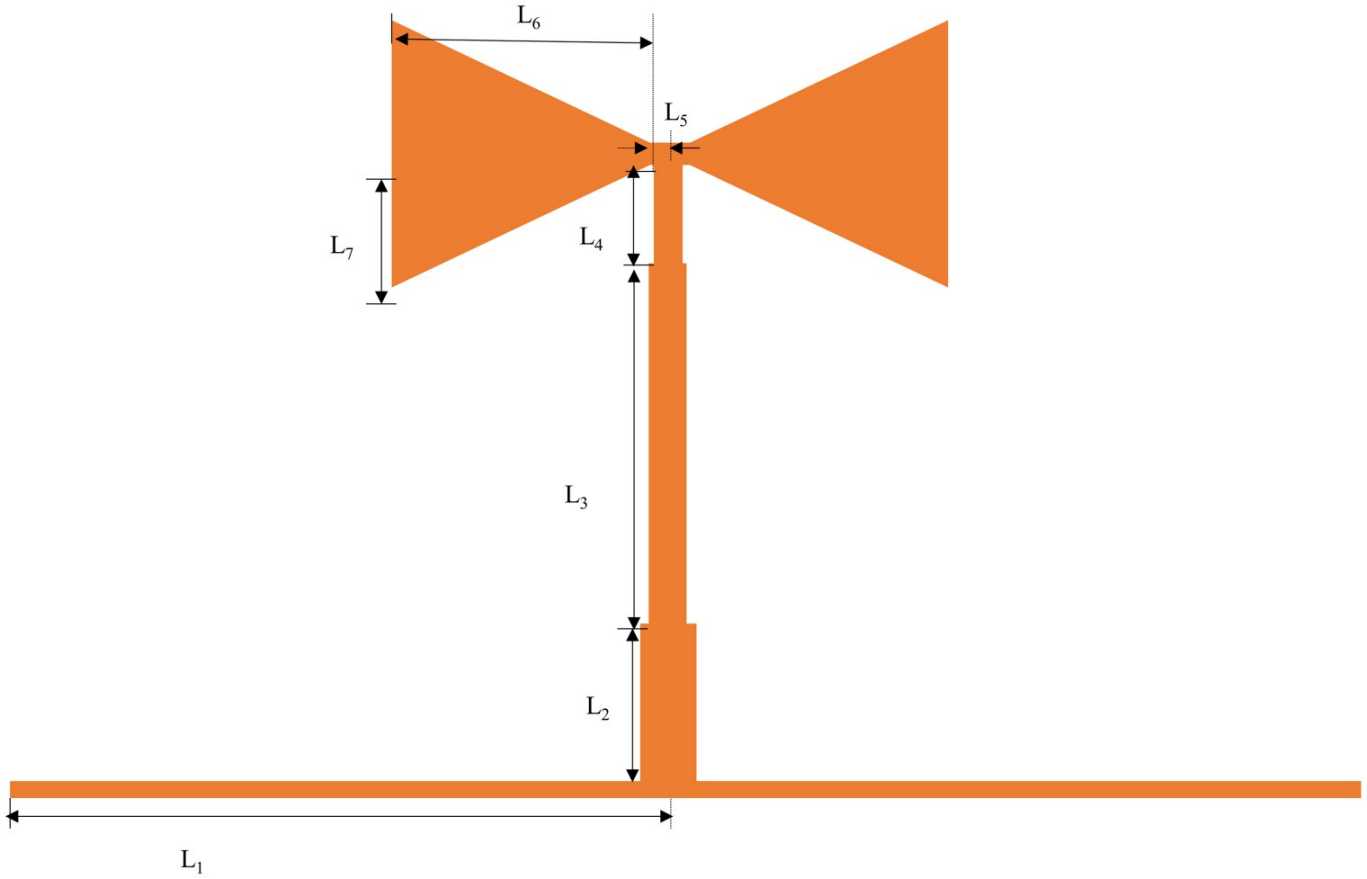
Stepped triangle resonator structure.

**Fig 4:**
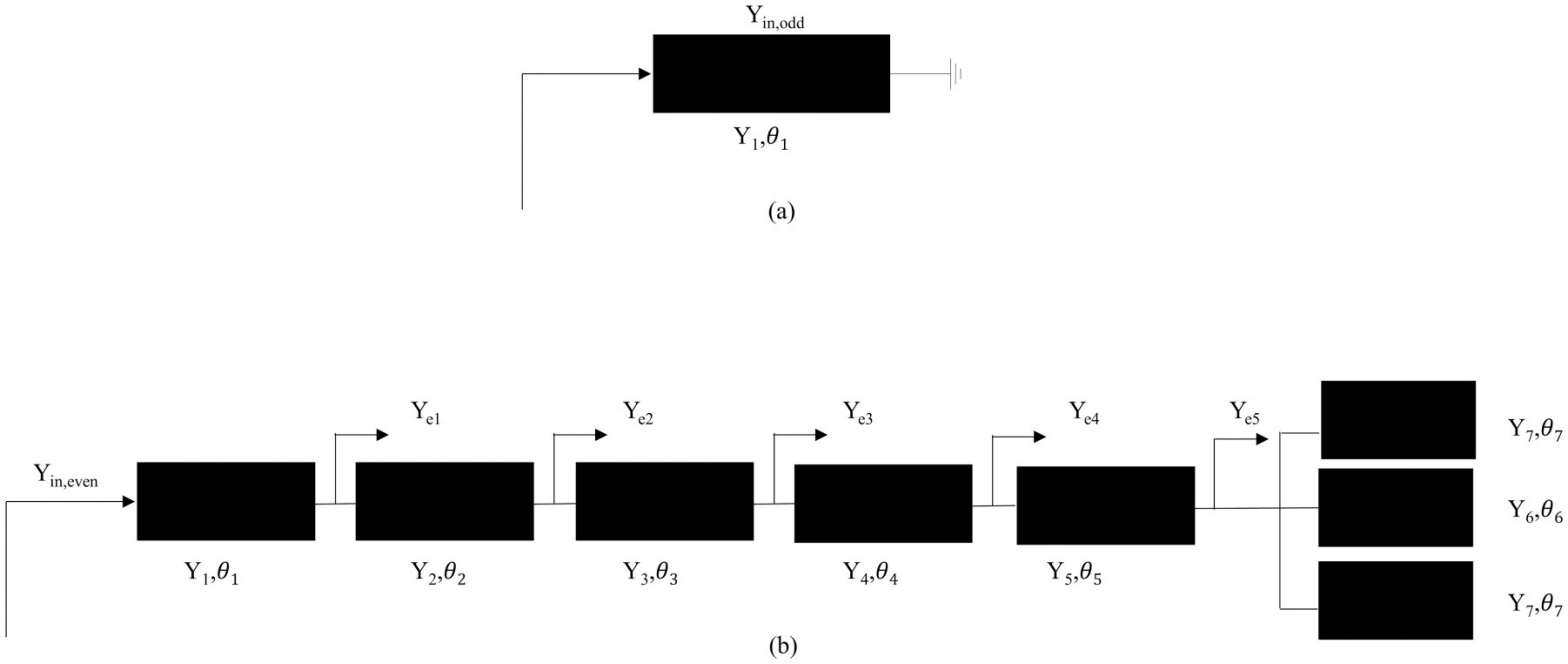
The odd and even mode structure of Stepped triangle resonator structure. (a) Odd-mode equivalent structures. (b) Even-mode equivalent structures.

Since this multimode resonator is symmetric about the axis T-T’, it can be studied and analyzed by the parity method. The multimode resonator and its parity mode equivalent structure are shown in Figs 3 and 4. The characteristic conductance, electrical length, and length of transmission of the horizontal transmission line, as well as the branches of each part of the stepped triangular multimode resonator, are denoted byand by *Y*,*θ*, and *L*.*Y*_*in*,*odd*_ and *Y*_*in*,*even*_ are the input conductances for odd and even modes,*Y*_*e*1_, *Y*_*e*2_, *Y*_*e*3_, *Y*_*e*4_, *Y*_*e*5_, *Y*_*e*6_ are the input conductances corresponding to each arrow, respectively. Fig 4(a) shows the equivalent structure of the resonator under odd-mode excitation; the symmetry plane T-T’ is short-circuited at this moment, and its odd-mode input conductance is expressed as:

$$\begin{array}{c} Y_{in,odd}=-jY_{1}cot\theta_{1} \end{array}$$ (2)

Fig 4(b) shows the equivalent structure of the resonator under dipole mode excitation, the symmetry plane T-T’ is a short open-circuit state at this moment, and the dipole mode input conductance is expressed according to the transmission line theory:

$$\begin{array}{c} Y_{in,even}=Y_{1}\frac{Y_{e1}+jY_{1}tan\theta_{1}}{Y_{1}+jY_{e1}tan\theta_{1}} \end{array}$$ (3)

Among them:

$$\begin{array}{c} Y_{e1}=Y_{2}\frac{Y_{e2}+jY_{2}tan\theta_{2}}{Y_{2}+jY_{e2}tan\theta_{2}} \end{array}$$ (4)

$$\begin{array}{c} Y_{e2}=Y_{3}\frac{Y_{e3}+jY_{3}tan\theta_{3}}{Y_{3}+jY_{e3}tan\theta_{3}} \end{array}$$ (5)

$$\begin{array}{c} Y_{e3}=Y_{4}\frac{Y_{e4}+jY_{4}tan\theta_{4}}{Y_{4}+jY_{e4}tan\theta_{4}} \end{array}$$ (6)

$$\begin{array}{c} Y_{e4}=Y_{5}\frac{Y_{e5}+jY_{5}tan\theta_{5}}{Y_{5}+jY_{e5}tan\theta_{5}} \end{array}$$ (7)

According to the resonance condition *Im*(*Y*_*in*_) = 0, when resonating, let Eqs (1) and (3) in Y_*in*,*odd*_ = Y_*in*,*even*_ = 0, it can be obtained that the odd mode resonant frequency satisfies *cotθ*, exactly, when *θ*_1_ = (2*n* − 1)*π*/2, included among these, are its solution and satisfie the resonance condition. At that time, its resonant frequency is:

$$\begin{array}{c} f_{1}=\frac{\text{c}}{4{\text{L}}_{1}\sqrt{\varepsilon_{\text{eff}}}} \end{array}$$ (8)

In the above equation: represents the speed of light in vacuum; represents the effective dielectric constant of the substrate. From the above equation, it can be seen that the resonant frequency of the odd mode is only related to the magnitude of the, The length of the central branch *L*_2_,*L*_3_,*L*_4_,*L*_5_,*L*_6_ does not have any effect on the odd mode resonant frequency. Let *K*_1_ = *Y*_1_/*Y*_2_,*K*_2_ = *Y*_2_/*Y*_3_,*K*_3_ = *Y*_3_/*Y*_4_,*K*_4_ = *Y*_4_/*Y*_5_;in addition,*Y*_5_ = *Y*_6_ = *Y*_7_,*θ*_5_ = *θ*_6_ = *θ*_7_, then the even-mode resonance frequency is computed by simplification as:

$$\begin{array}{c} \text{A}+{\text{jK}}_{2}tan\theta_{2}+{\text{jK}}_{1}tan\theta_{1}\left({\text{K}}_{2}+\text{jA}tan\theta_{2}\right)=0 \end{array}$$ (9)

In this:

$$\begin{array}{c} \text{A}=\frac{\text{B}+{\text{jK}}_{3}tan\theta_{3}}{{\text{K}}_{3}+\text{jB}tan\theta_{3}} \end{array}$$ (10)

$$\begin{array}{c} \text{B}=Y_{4}\frac{\text{C}+{\text{jK}}_{4}tan\theta_{4}}{{\text{K}}_{4}+\text{jC}\;\text{tan}\;\theta_{3}} \end{array}$$ (11)

$$\begin{array}{c} \text{C}=Y_{5}\frac{\text{j}4tan\theta_{5}}{1-3{\left(\text{tan}\;\theta_{3}\right)}^{2}} \end{array}$$ (12)

**Fig 5:**
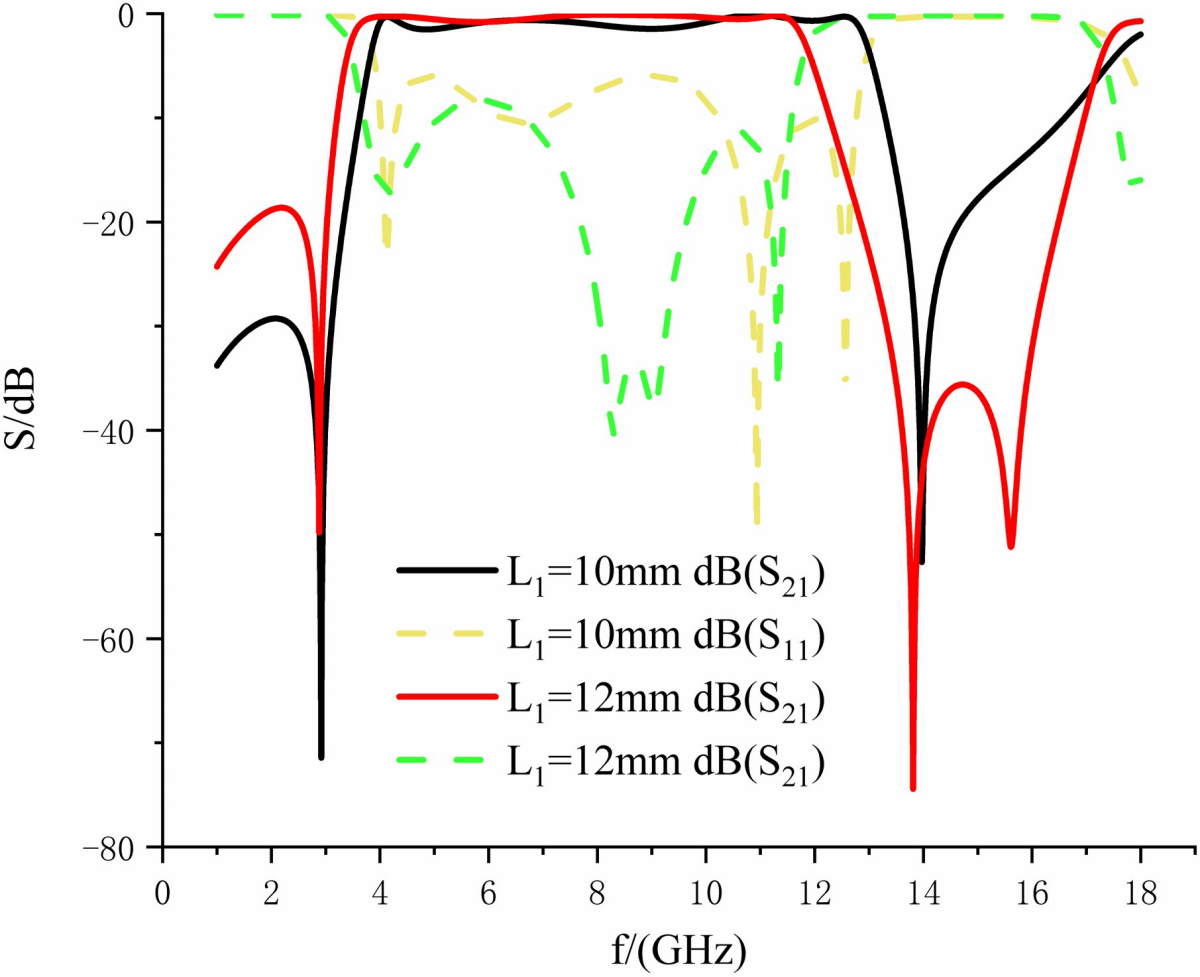
The influence of coupling line *L*_1_ on passband performance.

In the above equation:*θ*_1_ = *βL*_1_,*θ*_2_ = *βL*_2_,*θ*_3_ = *βL*_3_,*θ*_4_ = *βL*_4_,*θ*_5_ = *βL*_5_,*β* is the phase constant. From Eqs (1) to (12) above, the five resonant modes of the multimode resonator can be obtained. From Eqs (1) and (2), it can be seen that the resonator’s length mainly affects the odd mode’s resonance frequency. From Eqs (3) to (12), it can be seen that the resonance frequencies of the even modes are controlled by the entire multimode resonator. Meanwhile, the odd-mode resonant frequency *f*_1_ can be obtained algebraically from the above equation. To get the resonance frequencies of the odd modes, and then control the resonance frequency ranges by adjusting the resonator’s parameters, which This allows the design to have a more comprehensive frequency range and at the same time more freedom and flexibility. Eqs (8) and (9) reveal that as *L*_1_ increases, *f*_1_ shifts towards lower frequencies. By controlling the length of coupling line *L*_1_, the coupling strength can be regulated. From Fig 5, it can be observed that with increasing *L*_1_, the coupling between the input-output feed lines and the resonator continuously strengthens. Consequently, the entire passband shifts towards lower frequencies, with reduced insertion loss and increased return loss within the passband, and superior out-of-band suppression on the right side.

**Fig 6:**
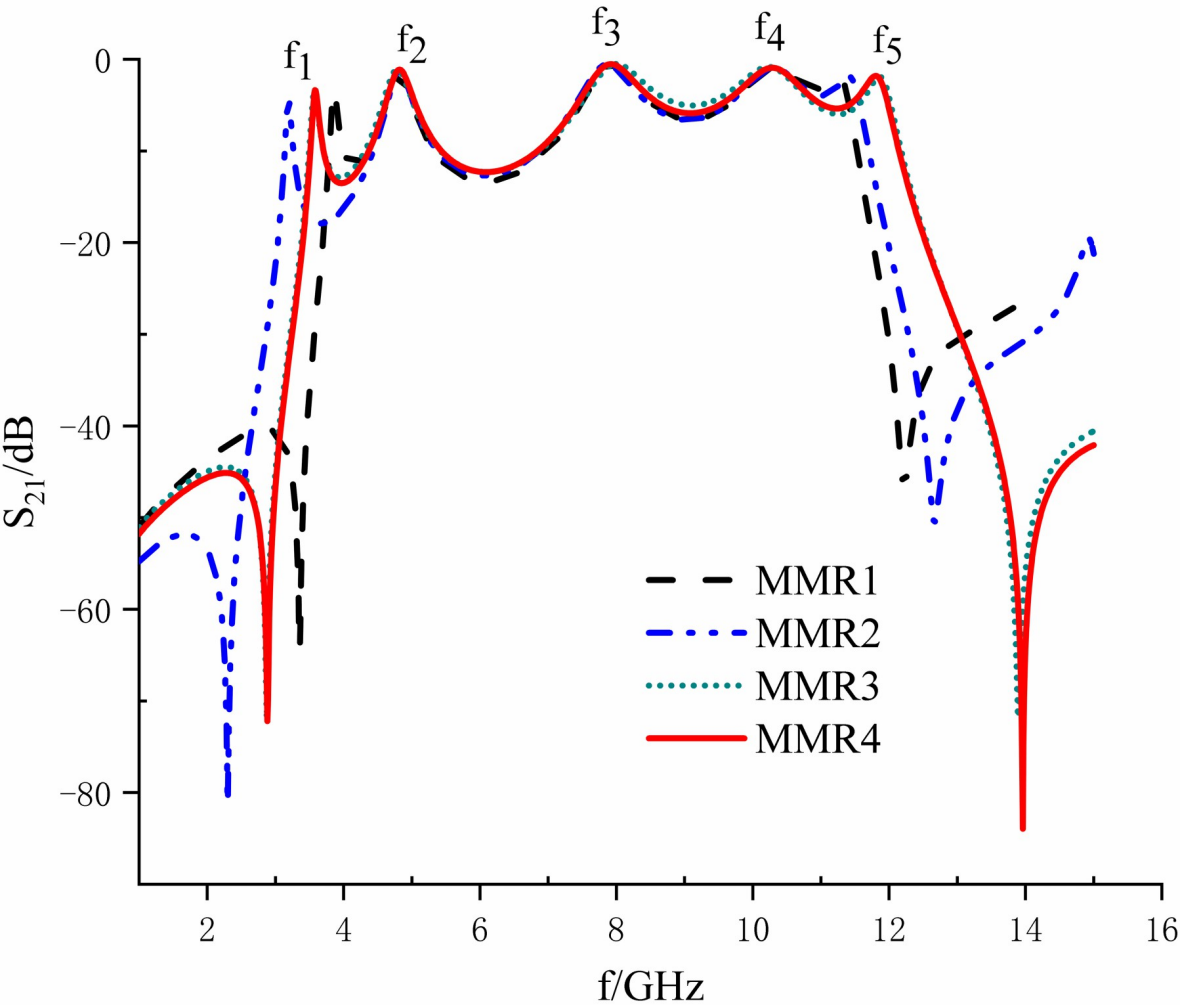
Curves of four MMRs under weak coupling.

According to the evolution process of the step-triangle type multimode resonator proposed in the previous section, the four types of MMRs are simulated separately to obtain the variation rule of the resonance point, as shown in Fig 6. From the observation of Fig 6: it can be obtained that compared with the traditional T-type structure, the resonance points of the other three improved multimode resonators are shifted to the low-frequency direction, which makes the ultra-wideband range more significant. As the structure of the MMR goes from simple to complex, the degree of out-of-band suppression deepens. In contrast, the triangular structure can further reduce the impedance discontinuity and offset compared to the rectangular one, significantly improving the dimensionality and the degree of freedom of adjusting the bandwidth.

In summary, based on the principle of a more comprehensive ultra-wideband range as well as more robust out-of-band rejection, the MMR4 is finally selected as the new multimode resonator, and the resonator is used to cross-toe coupled with the input/output feeder to form an ultra-wideband filter.

### 2.2 Design and simulation of UWB filte

**Fig 7:**
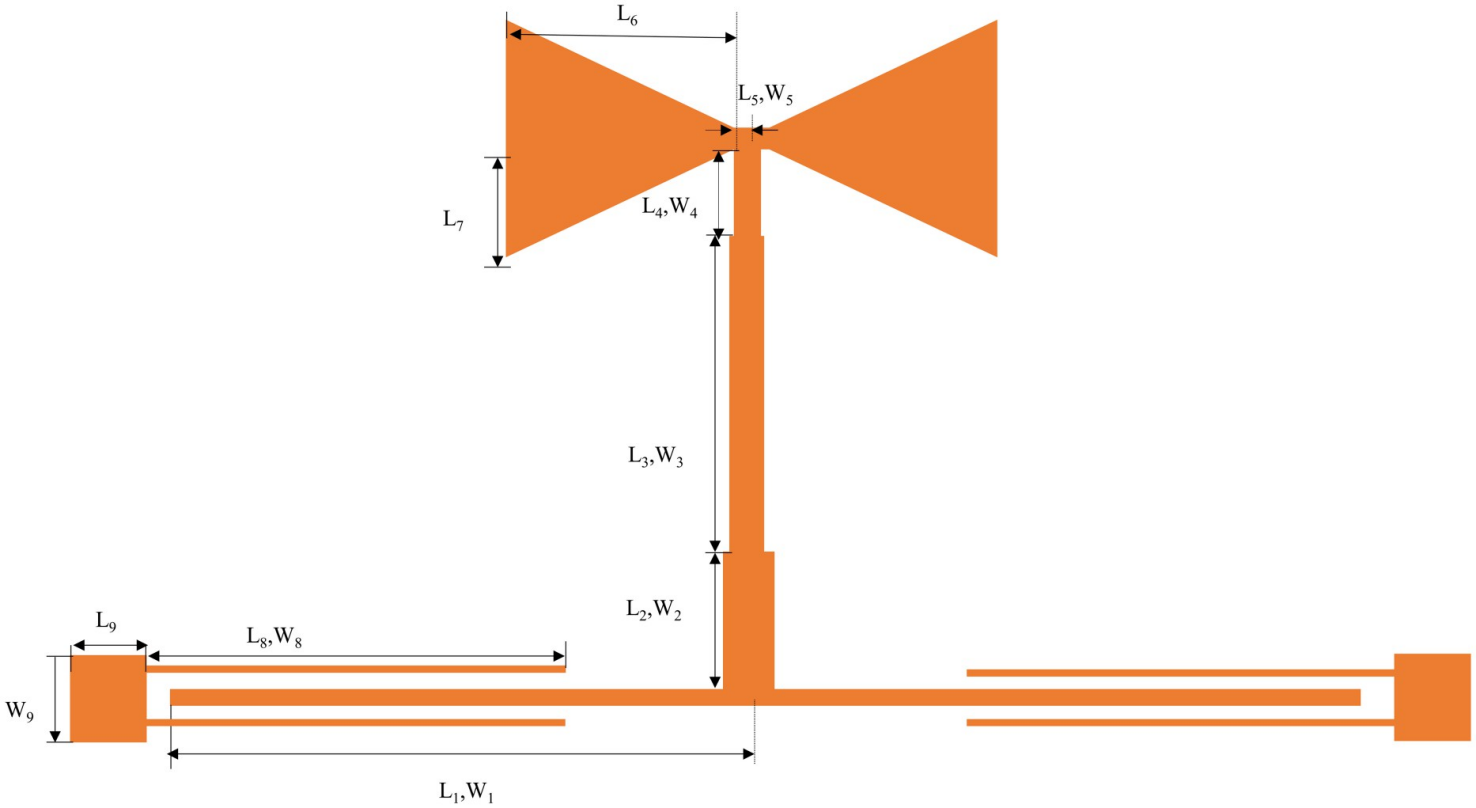
Schematic structure of ultra-wideband filter.

**Fig 8:**
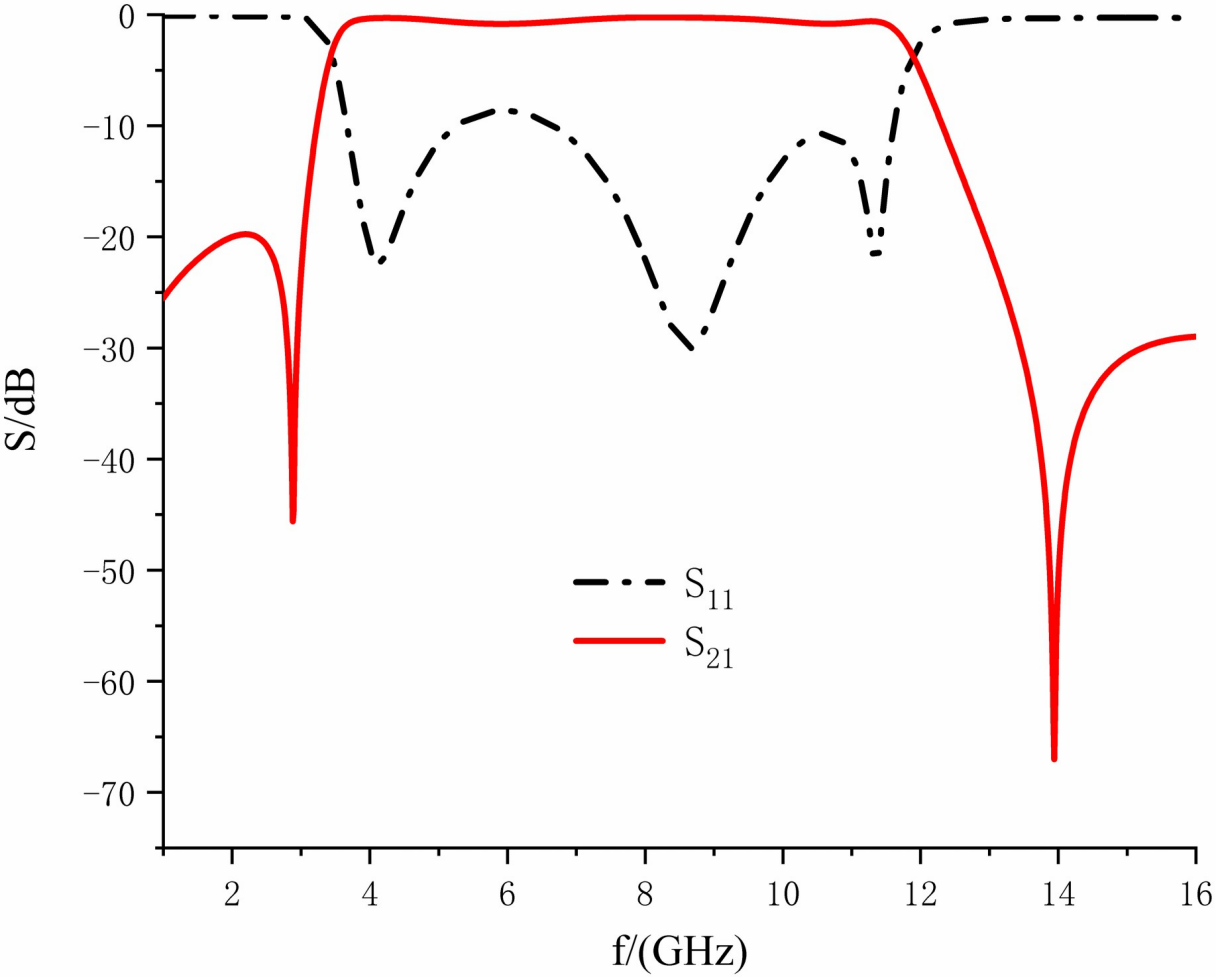
S-parameter simulation results of ultra-broadband filter.

The design of the ultra-wideband is realized by coupling the resonator to the input and output feeders, and the overall structure of this filter is shown in Fig 7. In this manuscript, HFSS15.0 is utilized for simulation. Fig 8 shows the S-parameter simulation results, where the insertion loss represents the return loss. From Fig 8, we know that the 3dB bandwidth of the ultra-wideband filter is in the range of 3.47 to 11.82GHz, the fluctuation of the passband is slight, and the insertion loss in the passband is less than 0.7dB, the return loss is within -8.60dB. The attenuation at 2.88 and 13.94 GHz reaches 45.61 dB and 67.03 dB, separately, with exceptional out-of-band rejection capability and overall performance.

The UWB filter designed in this manuscript uses a dielectric substrate RogersRT/duroid6006, relative permittivity 6.15, its loss angle tangent is 0.0019, the substrate thickness is 0.635mm, after repeated debugging and optimization to obtain the ultra-wideband filter structural parameters as Table 1.

**Table 1:** Structural parameters of UWB BPF (mm).

symbol	value	symbol	value	symbol	value	symbol	value
*W*_1_	0.15	*L*_2_	1.4	*W*_4_	0.2	*L*_5_	0.4
*L*_1_	6	*W*_3_	0.3	*L*_4_	1	*L*_6_	2.37
*W*_2_	0.48	*L*_3_	3.3	*W*_5_	0.2	*L*_7_	1.25
*W*_8_	0.1	*L*_8_	5.5	*W*_9_	1.4	*L*_9_	1.6

## 3 Dual notch UWB filter design with advanced selectivity

### 3.1 Dual notch UWB filter design

Due to the broad spectrum encompassed by Ultra-Wideband (UWB) systems (3.1–10.6 GHz), numerous narrowband communication systems coexist within this range. In order to avoid the influence of these narrowband signals on ultra-wideband systems, the design of ultra-wideband filters with stopbands performance has gradually become the focus of scholars’ research [12]. There are many ways to design the stopband filter, such as coupled resonator structure, asymmetric coupling line, parallel open-circuit branch, etched DGS structure. Among them, the coupled resonator structure and asymmetric coupling line method are simple and convenient. They can achieve a deeper stopband characteristic without expanding the original size, which meets the demand for miniaturization and has less impact on the original passband.

**Fig 9:**
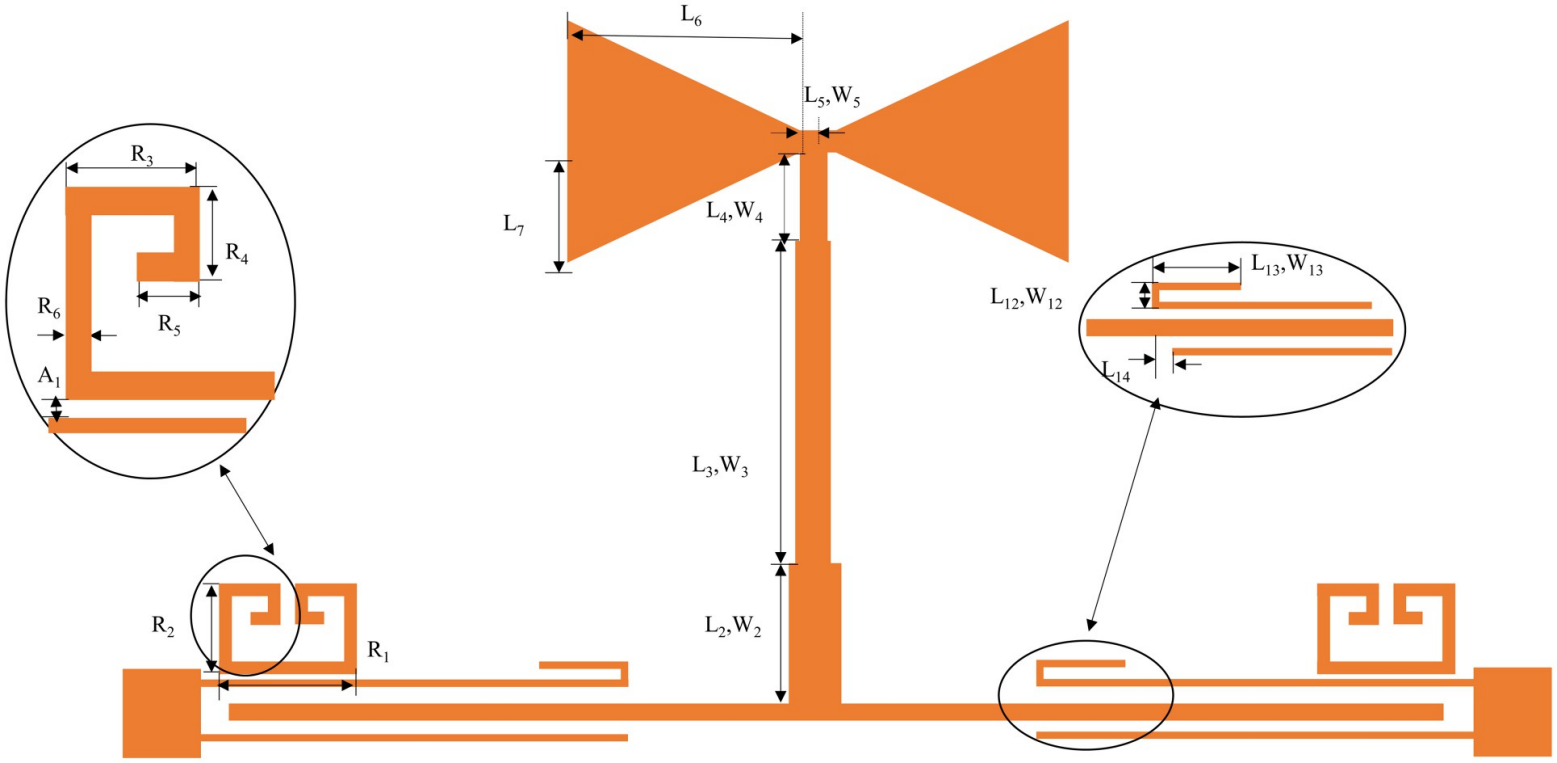
UWB filter structure with double-notch bands.

**Fig 10:**
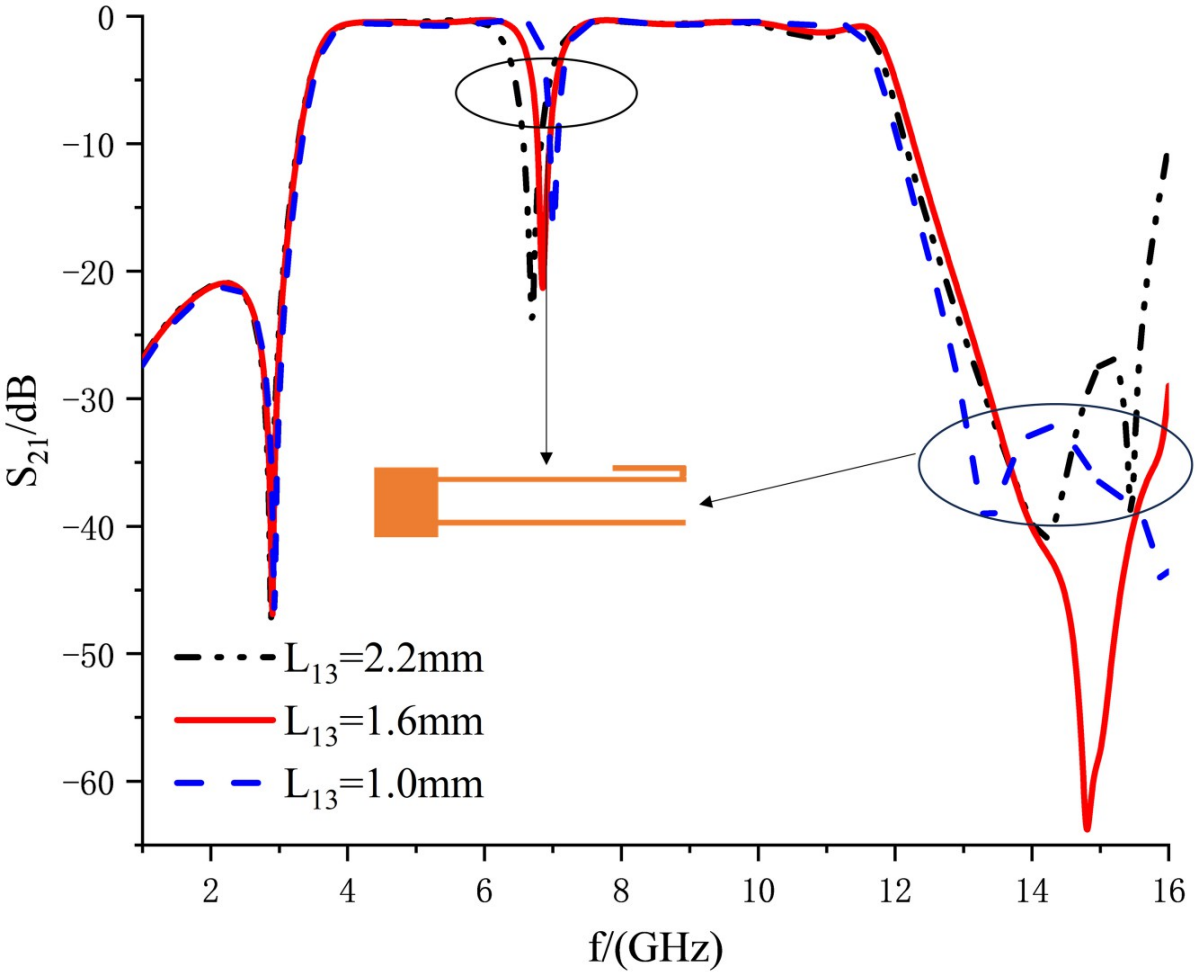
Effect of *L*_13_ on notch frequency.

**Fig 11:**
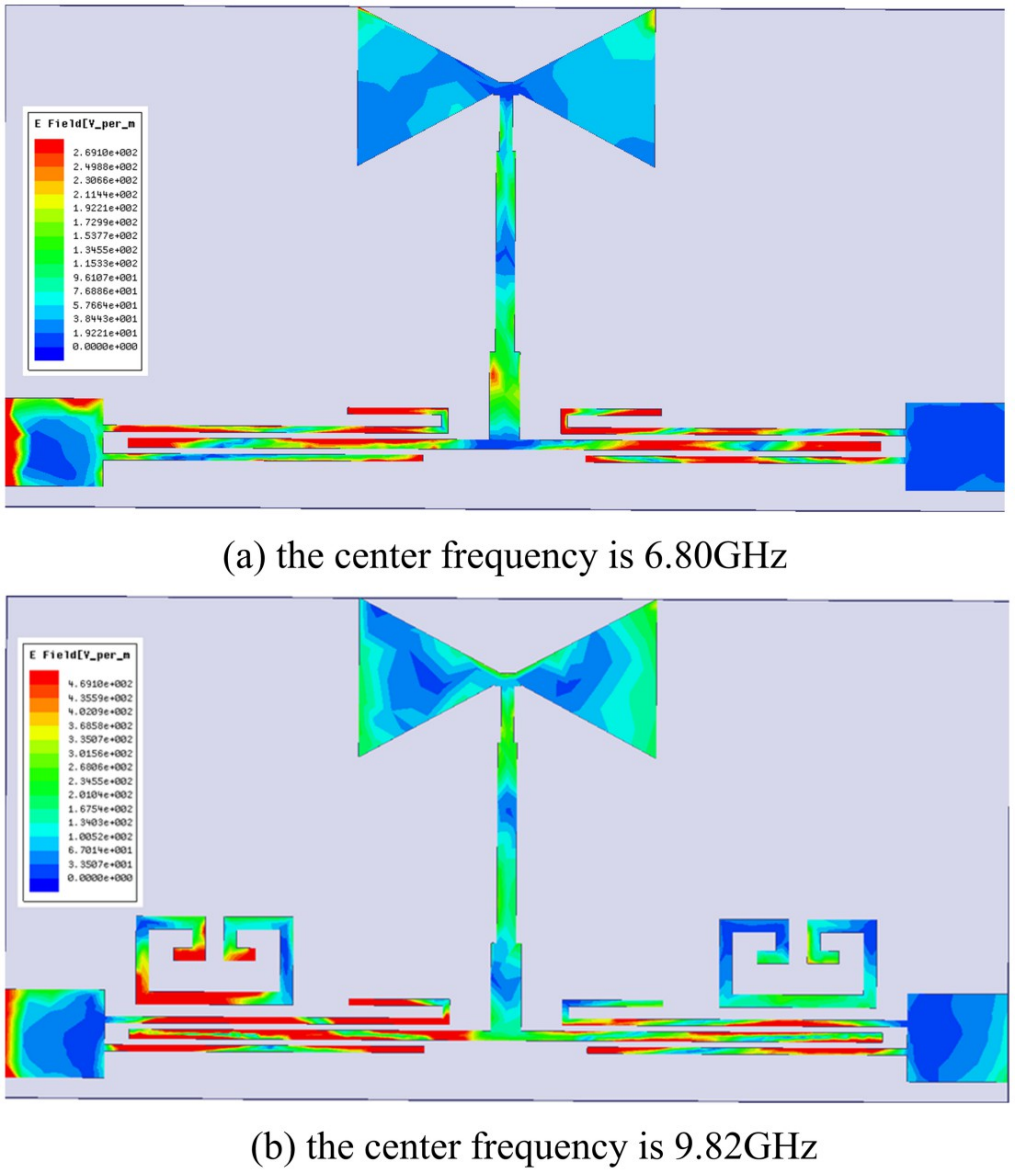
Surface current distribution diagram. (a) the center frequency is 6.80GHz. (b) the center frequency is 9.82GHz.

Based on this, this work proposes a compact dual-notch bands UWB filter. Further processing of the previously designed UWB filter, through the asymmetric coupling line with the multimode resonator coupled on both sides of the other side of the split ring resonator (SRR), introduces the two notches; the specific structure is shown in Fig 9. The first notch is created by the asymmetric coupling method, the structure of which is shown in the enlarged view on the right side of Fig 9. The structure consists of extending the upper feeder and bending it into a U-shape, with the bottom being *L*_12_ in length and *L*_13_ in height and the lower feeder shrinking inward by a distance of *L*_14_. By changing the height *L*_13_ of the U-shape, the position and depth of the first notch can be adjusted. The U-shaped high *L*_13_ takes the values of 0.4, 1.6, and 2.8 mm. The simulation results depict the first notch frequency variation curve ranging from 6.2 to 7.54 GHz, as illustrated in Fig 10. Keeping the dimensions of other branches fixed, altering only the value of *L*_13_ induces a continuous shift in the center frequency of the notch. Upon observation, it is noted that with the increase in *L*_13_, the center frequency of the first notch progressively shifts towards the lower frequencies. Consequently, independent control over the position of the first notch frequency can be achieved by adjusting the length of *L*_13_, thereby mitigating interference in specific frequency bands. Several Wireless Local Area Network (WLAN) systems operate near the 6.80 GHz frequency band. By introducing a notch structure, interference from these systems can be filtered out, thus enhancing the performance and reliability of communication systems. When the center frequency of the notch is set to 6.80 GHz, Fig 11(a) reveals a dense current distribution in the asymmetrical branch region. In contrast, the current distribution in other areas is sparse. This confinement of energy within the structure facilitates the realization of notch characteristics, enabling the suppression of signals within this narrowband frequency range.

In order to introduce the second notch, a loaded SRR is used coupled to both sides of the multimode resonator, whose structure and parameter settings and parameter settings are shown in the left enlargement of Fig 9. The resonator is constructed by folding a half-wavelength uniform transmission line. Resonance occurs at *θ*_*T*_ = *n*Π, where *n* = 1. The resonance frequency expression for this SRR is provided by:

$$\begin{array}{c} f_{m}=\frac{c}{2(R_{1}+R_{2}+R_{3}+R_{4}+R_{5})\sqrt{\varepsilon_{eff}}} \end{array}$$ (13)

**Fig 12:**
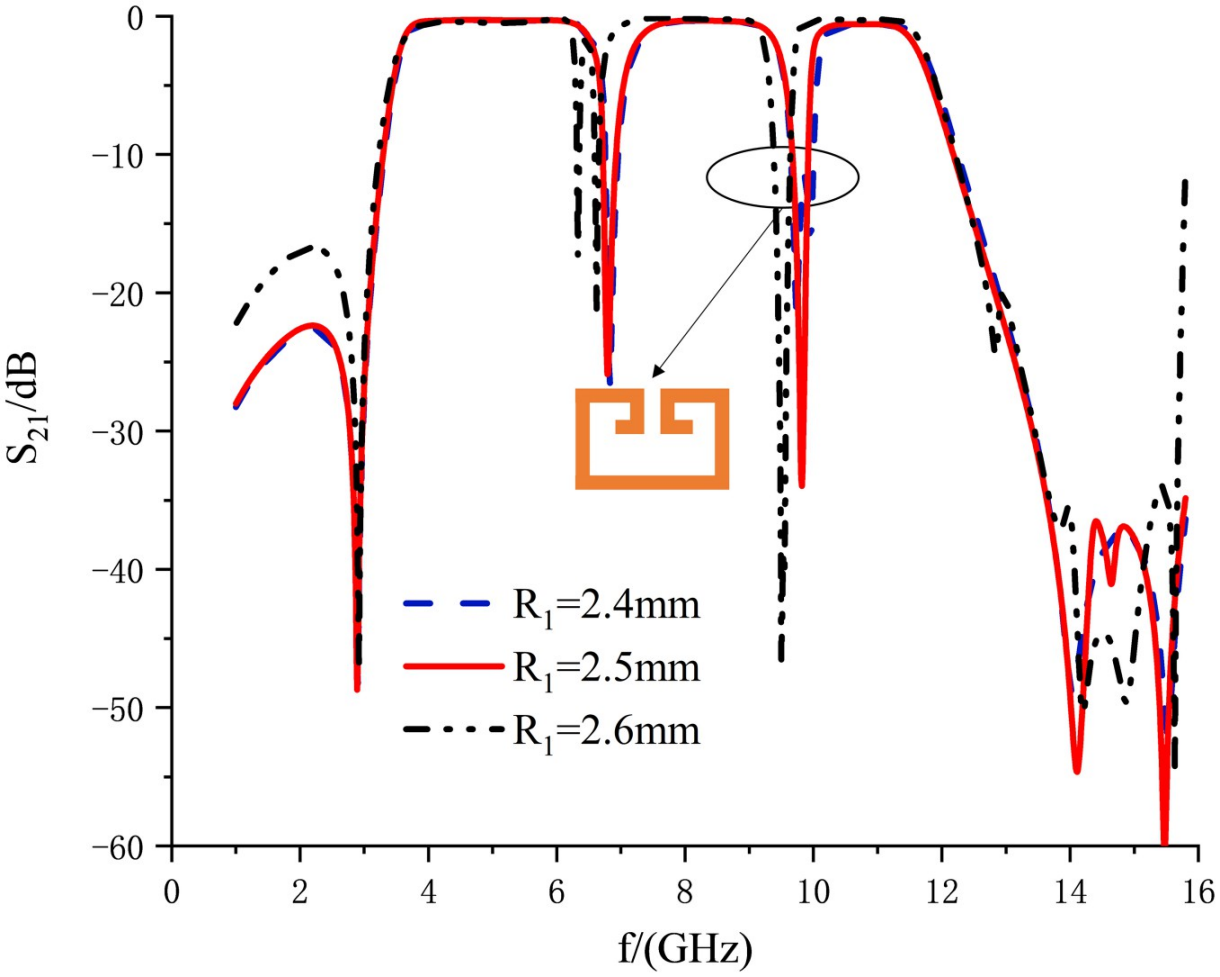
Effect of *R*_1_ on notch frequency.

It is evident from the above formula that the resonance frequency varies inversely with the physical length of the resonator and the dielectric constant of the substrate. Fig 12 illustrates the influence of *R*_1_ on the notch frequency. Taking into account the depth and range of the first notch and the position of the second notch, *R*_1_ is ultimately selected as 2.5mm. To mitigate interference from the X-band satellite link frequency range (9.6-12.3GHz) and enhance passband performance, a notch is introduced at 9.82GHz. Examining the current distribution in Fig 11(b) at a frequency center of 9.82GHz, the current predominantly concentrates at the SRR, and energy concentrated in the notch structure is unable to effectively transmit to the output feedline, thereby generating the notch. With an increase in *R*_1_, the center frequency of this notch shifts towards lower frequencies. Hence, notch frequency tunability can be achieved by adjusting *R*_1_.

### 3.2 Design with highly selective out-of-band rejection characteristics

Defected Ground Structure (DGS) represents a novel architectural concept introduced by the Korean scholar J.L. Park et al. in 1999 [13]. The precise etching of a specific shape on the ground plane [14] disrupts the distribution of ground plane currents, consequently modifying the characteristics of transmission lines. This alteration extends to the effective capacitance and inductance, conferring band-stop and slow-wave properties [15]. DGS’s development is rooted in photonic bandgap structures, sharing similarities with them in their ability to impede the transmission of electromagnetic waves within a designated frequency range, thereby exhibiting distinctive bandgap characteristics.

**Fig 13:**
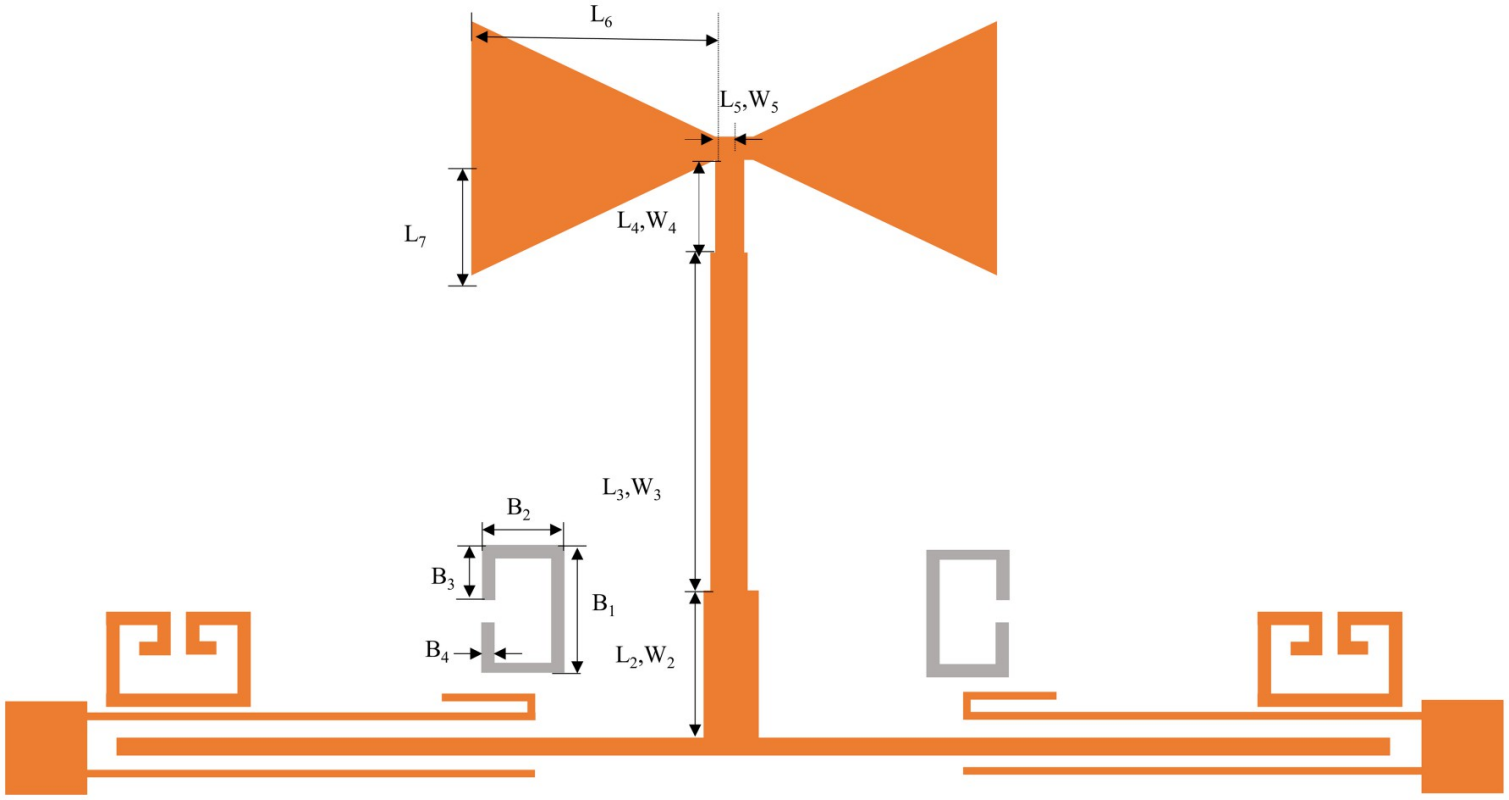
Dual-notch UWB filter with highly selective out-of-band rejection.

**Fig 14:**
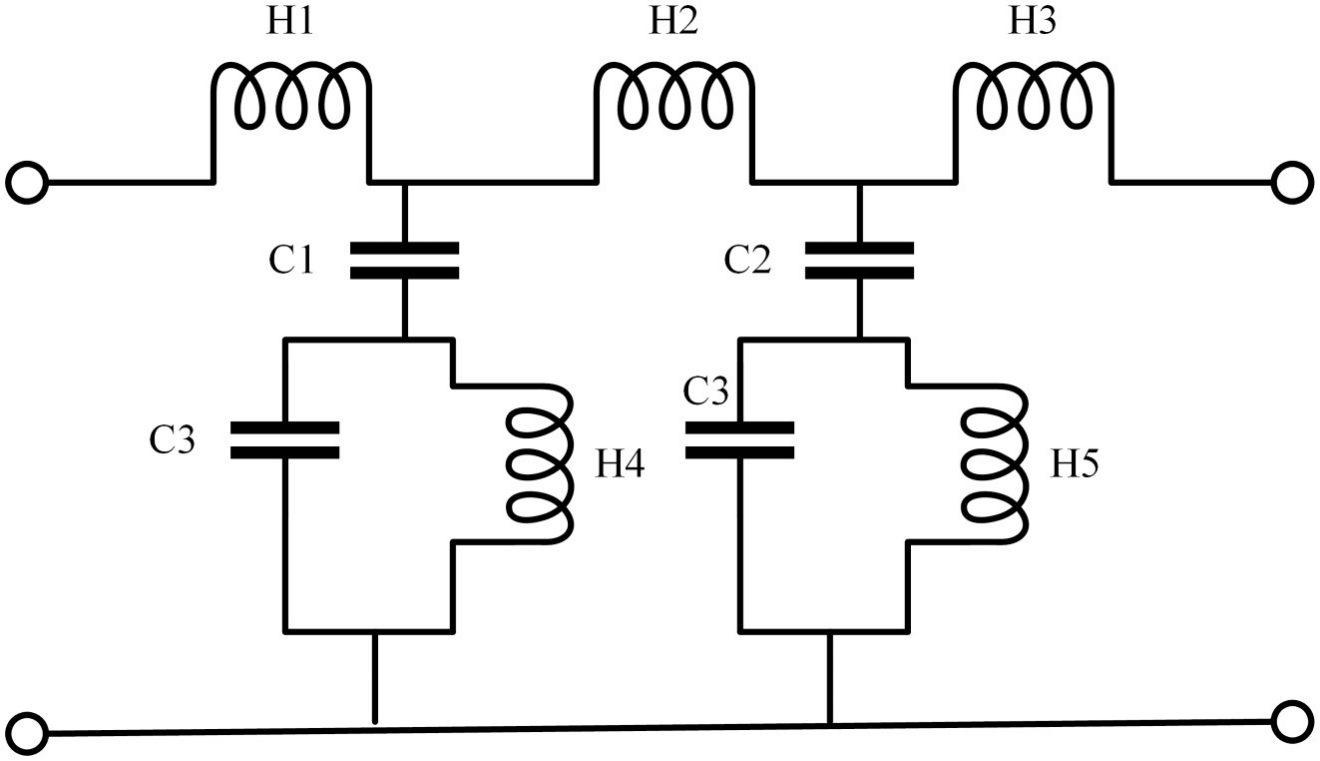
Equivalent circuit structure.

In this manuscript, a pair of C-type DGS SRR are etched on the ground plane, and then the proper transmission zero is introduced to improve the stopband characteristics of the filter. The specific structure is shown in Fig 13, illustrating a resonant unit comprising two symmetrically arranged open resonant rings. The equivalent circuit diagram of the DGS SRR is presented in Fig 14, showcasing a parallel resonator composed of *H*_1_ and *C*_1_ formed by the left and right resonant rings. Analyzing the frequency response reveals that, with a constant width *B*_4_ of the symmetrical open-ring resonator, the resonant ring’s equivalent inductance H1 primarily depends on the side length *B*_1_, while the equivalent capacitance *C*_1_ is chiefly determined by the opening length *B*_3_ of the resonator. Furthermore, an increase in the side length *B*_1_ of the resonant ring increases both the equivalent inductance *H*_2_ and the equivalent capacitance *C*_2_ of the transmission line. The resonance frequency of the C-shaped open resonant ring is determined by the resonance frequency *f*_*s*_ of the parallel branch, which comprises *H*_1_, *C*_1_, and *C*_2_. The expression for *f*_*s*_ is given by:

$$\begin{array}{c} f_{s}=\frac{1}{2\pi \sqrt{H_{1}\left(C_{1}+C_{2}\right)}} \end{array}$$ (14)

the capacitance and inductance of the resonant circuit can be calculated as:

$$\begin{array}{c} C_{1}=\frac{\omega_{0}}{Z_{0}g_{1}}\frac{1}{\omega_{0}^{2}-\omega_{c}^{2}} \end{array}$$ (15)

$$\begin{array}{c} H_{1}=\frac{1}{4\pi_{2}\pi f_{b}C} \end{array}$$ (16)

Where *f*_*b*_ represents the resonance frequency of the single-pole Butterworth low-pass filter; *w*_*c*_ denotes the 3dB attenuation angular frequency; *w*_0_ represents the load impedance, and *g*_1_=2 denotes the normalized inductance component value of the single-pole Butterworth low-pass filter. Therefore, controlling the length of *B*_1_ allows for the adjustment of the position of the right transmission zero, consequently enhancing the out-of-band transmission performance.

**Fig 15:**
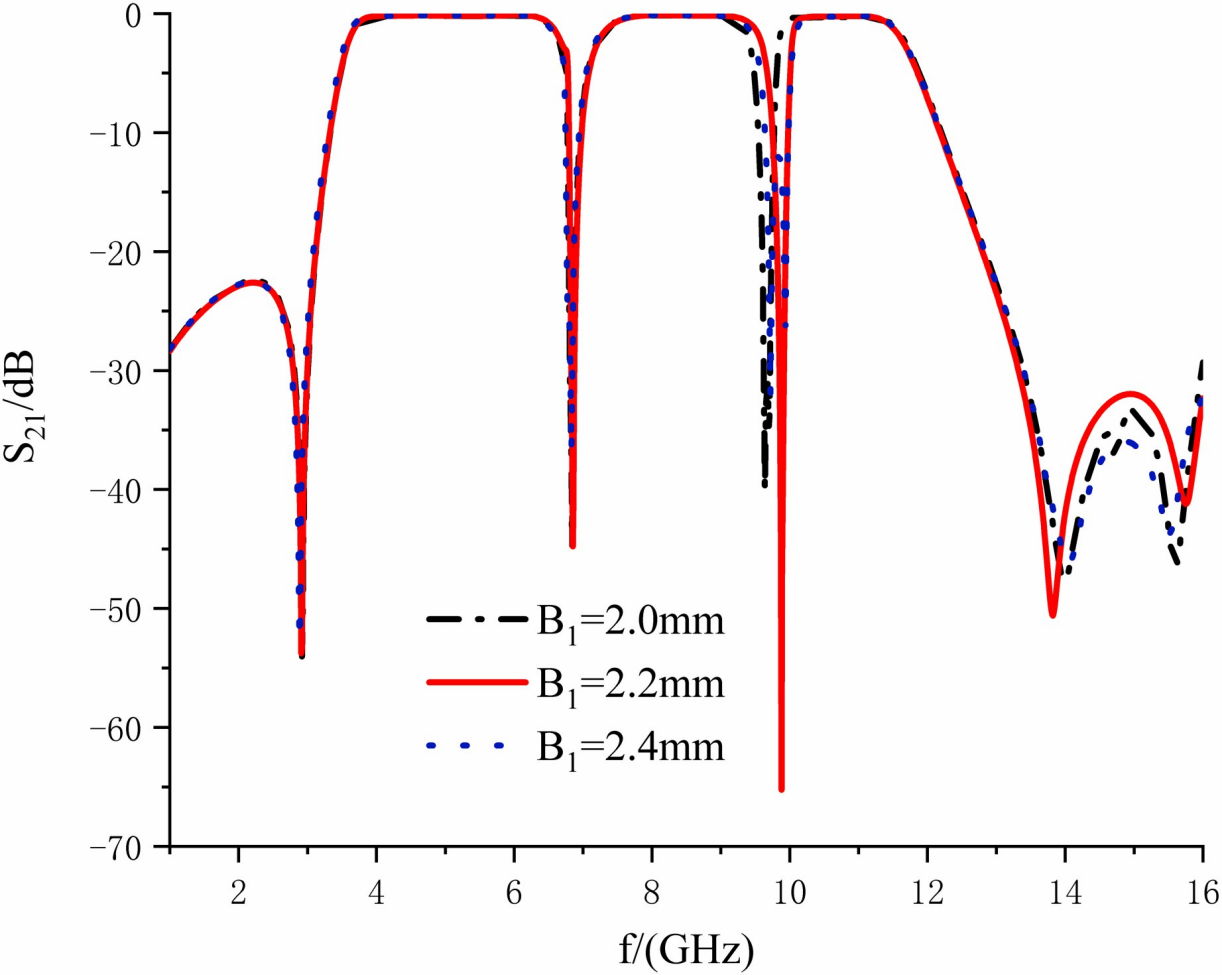
Effect of variation of *B*_1_ length on right transmission zero frequency.

Fig 15 shows the insertion loss curves when the lengths of *B*_1_ are taken as 1.8, 2.0, and 2.2mm, correspondingly. It can be observed that with the increasing length, the frequency of the second notch is shifted to the right. The depth of the web is increased, while the attenuation of the correct transmission null is enhanced, the out-of-band suppression ability is improved, and the introduction of the transmission null has steep passband edges. Moreover, it has minimal impact on the notch depth and frequency of the first one. Therefore, the length can be adjusted so that the correct transmission zero is located at 14.12 GHz, so that its attenuation reaches 54.52 dB, and the out-of-band attenuation is greater than 30 dB in 13.41-16.0 GHz, which is an effective suppression of the interference of parasitic signals.

**Fig 16:**
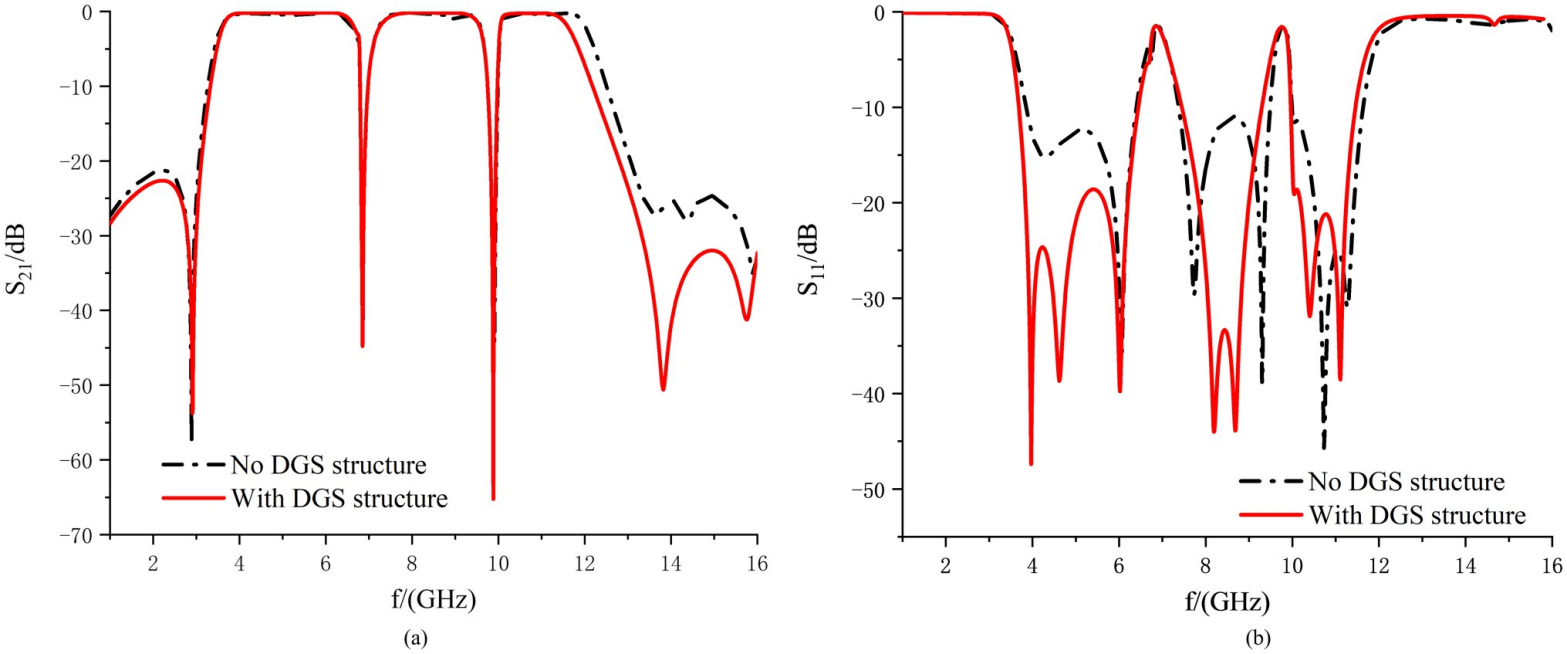
Comparison of structures with and without DGS.

From Fig 16(a), it can be seen that by introducing the DGS SRR structure, the correct transmission zero is deepened based on the original dual-notch bnads UWB filter so that the attenuation is increased from 32.29 dB to 54.52 dB, the parasitic passband is suppressed, and the lower rejection band is broadened. At the same time, the 30 dB attenuation rejection band is widened with almost no effect on the frequency and depth of the first notch, and the rejection capability is greater than 30 dB in the range of 13.41 to 16.0GHz range of suppression are greater than 30dB, which improves the attenuation in this frequency band, thus presenting a more desirable suppression performance. Meanwhile, from Fig 16(b), it can be seen that the introduction of this structure is equivalent to changing the position of the reference ground, changing the thickness of the medium, and compensating for the loss of the medium so that the return loss in the passband is improved from 10.96dB to all more remarkable than 18.61dB.

### 3.3 Simulation

**Fig 17:**
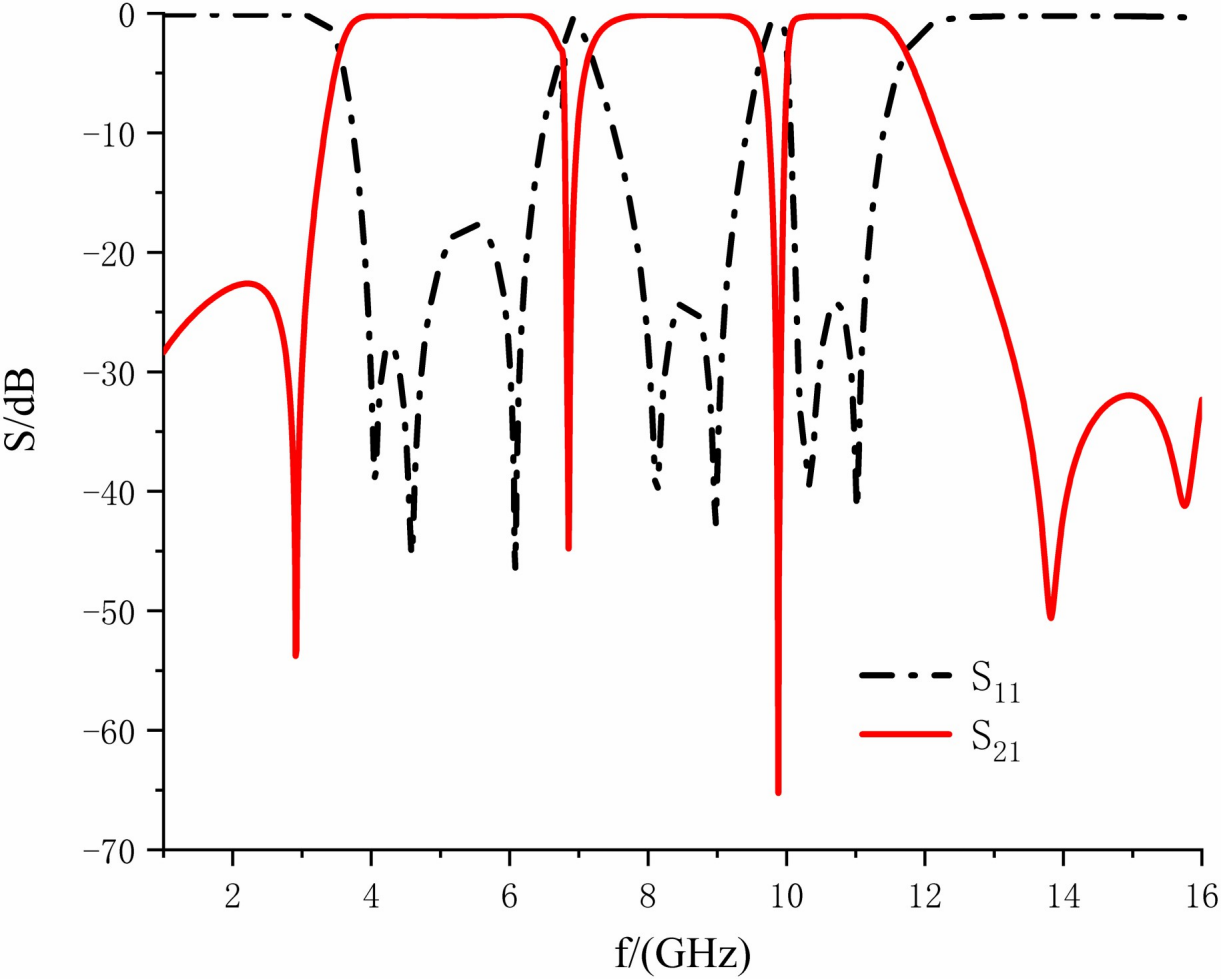
S-parameter simulation plot diagram.

**Fig 18:**
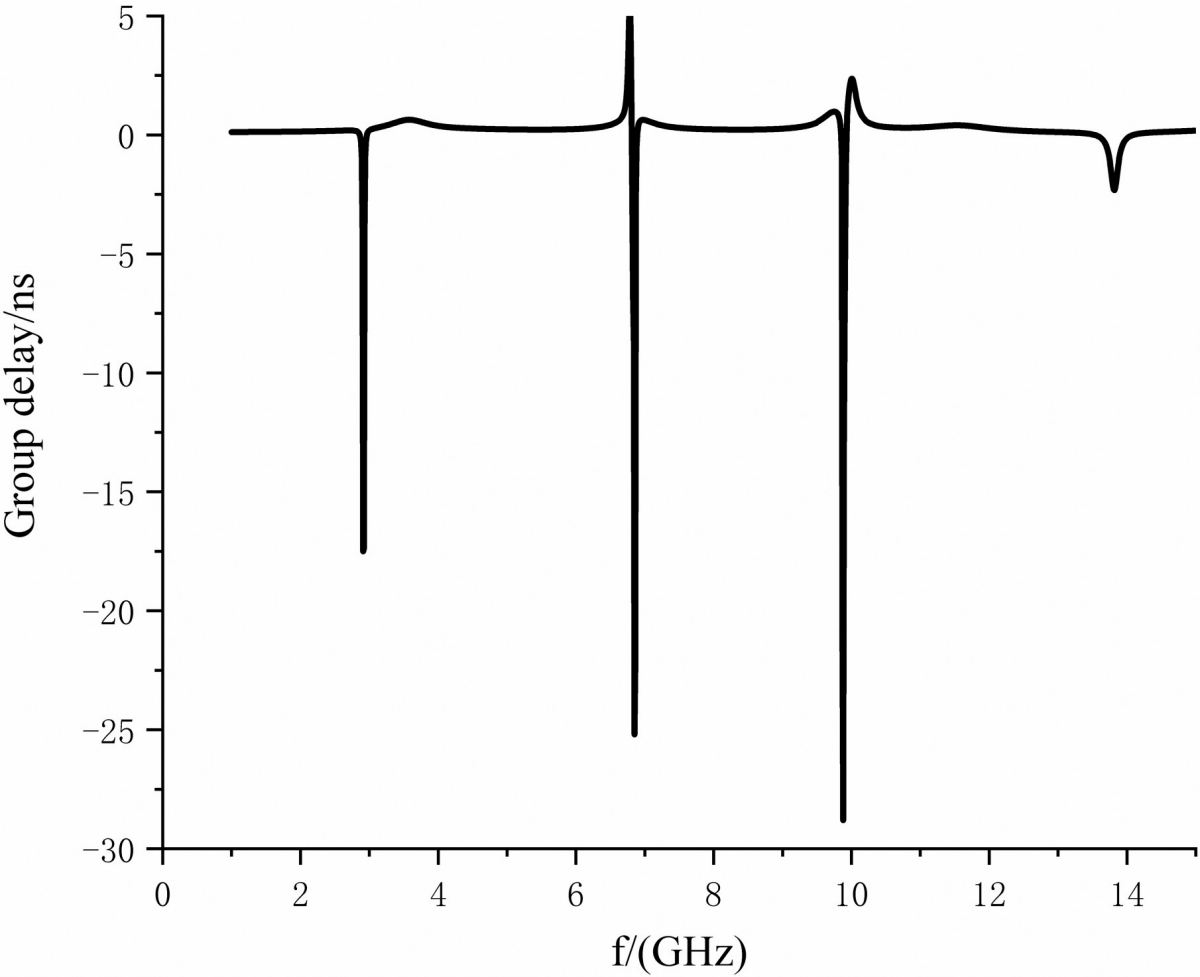
Group delay characteristic curve.

After the above analysis,Combining the center frequency position of the notch and the principle that the more profound the notch depth, the better, the steeper the passband edge introduced by the transmission zero, the better. Using of HFSS15.0 continuous testing and optimization, and ultimately, the two notches structure and the specific size of the DGS structure, as shown in Table 2. The simulation curves of return loss, insertion loss, and group delay characteristics are shown in Figs 17 and 18.

**Table 2:** Dimensional sizes of the two notch structures and the DGS SSR structure (mm).

symbol	value	symbol	value	symbol	value	symbol	value
*W*_12_	0.1	*L*_14_	0.4	*R*_4_	0.7	*B*_1_	2.2
*L*_12_	0.2	*R*_1_	2.5	*R*_5_	0.5	*B*_2_	1.4
*W*_13_	0.1	*R*_2_	1.4	*R*_6_	0.2	*B*_1_	0.9
*L*_13_	1.6	*R*_3_	1.1	*A*_1_	0.5	*B*_4_	0.2

As shown in Fig 17, the filter’s passband is 3.52-11.68 GHz, and the center frequency is 7.59 GHz. The in-band ripple is tiny, and the minimum insertion loss is only 0.61 dB; the return loss in the passband is greater than 18 dB. The transmission zero attenuation on both sides is more than 47 dB, and the out-of-band attenuation is greater than 30 dB within the range of 13.41-16.0 GHz, which reaches the goal of suppressing the parasitic passband and broadening the lower resistance band, with excellent out-of-band suppression performance. The rectangular coefficient of the filter is 1.34, which is exceptional for filtering out the interference signals in the parasitic passband with superior selectivity. Two notches are generated in the passband: the center frequency of the first notch is 6.80GHz, the 3db bandwidth is 6.61-7.14GHz with a depth of -26.21dB; the center frequency of the second notch is 9.82GHz, and the 3dB bandwidth is 9.48-10.0GHz, with a depth of -35.11dB. The essential performance of the filter is fantastic.

Fig 18 illustrates the group delay characteristic curve of the filter, representing the temporal delay magnitude as a signal traverses through it. The filter inherently introduces a delay to the signal, and the variation in delay at different frequency points indicates the degree of phase distortion of the signal after filtration. The specific definition of group delay is expressed as:

$$\begin{array}{c} \tau_{d}=\frac{\alpha \varphi_{T}}{\alpha \omega } \end{array}$$ (17)

where *φ*_*T*_ denotes the insertion phase shift, and *ω* represents the angular frequency. From Fig 18, it is evident that there is a sharp increase observed within the frequency bands of the two notches and at the passband cutoff frequency. Within other regions of the passband, it remains relatively stable, with values less than 0.24 ns, indicating a fundamentally stable state. The abrupt increases occur at four distinct points: the first and fourth points correspond to transmission zeros, while the second and third correspond to the two notch frequencies. At these points, there is significant jitter in the group delay, whereas the remainder of the passband essentially maintains stability.

## 4 Testing and verification

**Fig 19:**
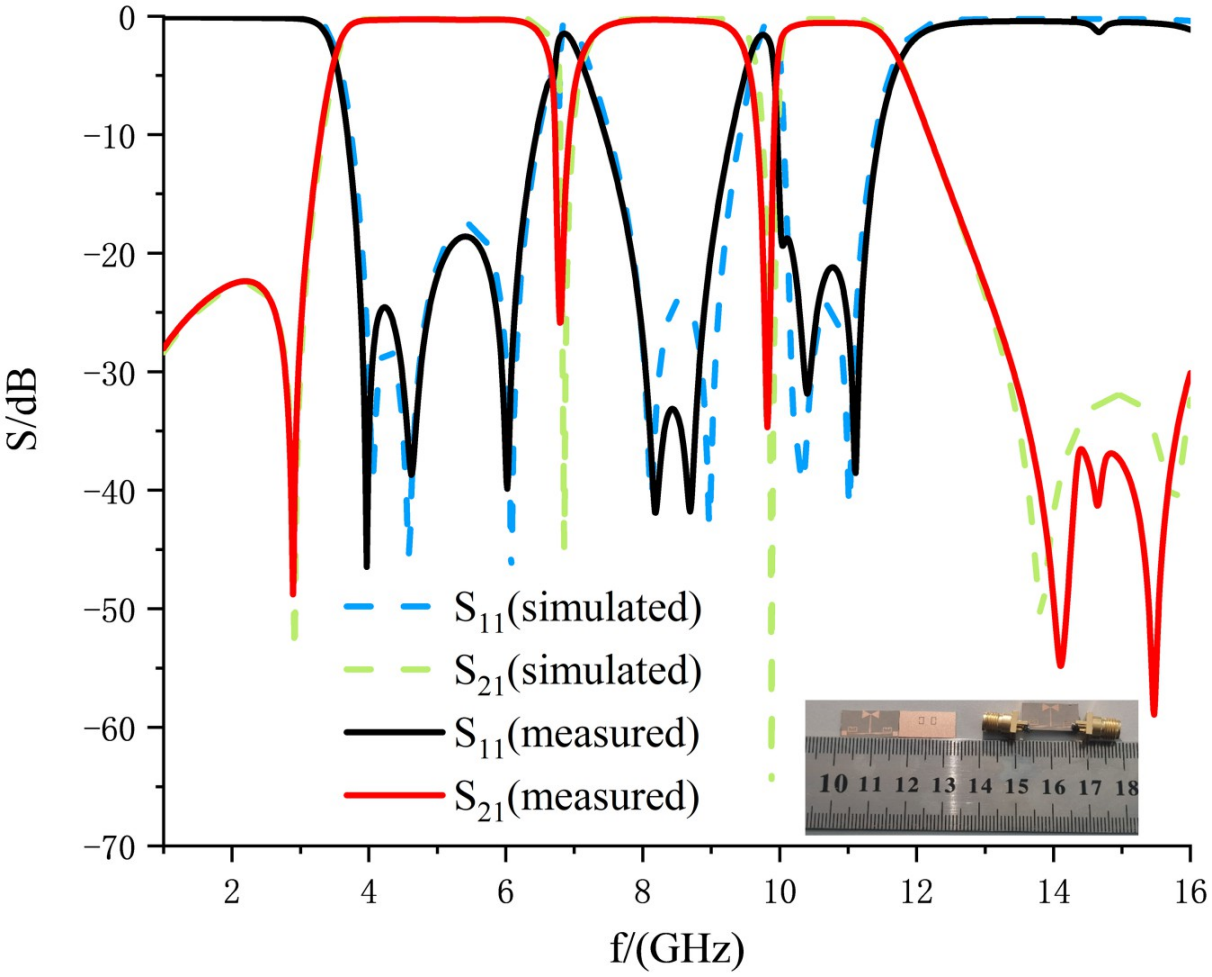
Comparison between test results and simulation results.

The physical fabrication of the filter is carried out by combining the dimensional parameters of the filter given in Tables 1 and 2. The front view and back view of the physical filter are shown in the lower side of Fig 15. This ultra-wideband filter designed in this manuscript uses Rogers RT/duroid 6006 dielectric substrate with a relative permittivity of 6.15, loss tangent angle of 0.0019, substrate thickness of 0.635mm, and size of just 0.41λ_*g*_×0.20λ_*g*_. The physical filter is tested using a vector network analyzer Agilent N5247A, and the test results are compared and analyzed with the simulation results. The results are compared and analyzed, and the results are shown in Fig 19. From Fig 19, it can be observed that the test results have the same trend as the simulation results, but there are some minor deviations. There are many factors leading to the departure of the results; on the one hand, it may be due to the small size of the microstrip line, which is inevitably deviated due to the limitation of the fabrication process and processing accuracy; on the other hand, it may be due to the parasitic effect of the soldered RF SubMiniature version A (SMA) joints.

Table 3 shows the performance comparison between the ultra-wideband filters designed in this manuscript with dual notch characteristics and the reference [2, 8, 16–27]. From Table 3, it can be seen that the ultra-wideband filter designed in this document has an extensive and smooth passband range and superb passband performance; the notch characteristics are better, with notch depths of more than 27 dB; the introduction of the left and right transmission zeros has steep passband edges and superior out-of-band rejection; and it has a significant advantage in terms of size to meet the demand for miniaturization in modern circuit design.

**Table 3:** Comparison of filter parameters between this work and other filters.

Ref.	IL(dB)	RL(dB)	Passband(GHz)	NB(HGz)/Attenuation(dB)	Attenuation at TZs(dB)	GD/ns	Size(λ_*g*_×λ_*g*_)
[16]	<0.5	>10	3.05-10.8	Not available	>30,>30	NA	0.61×0.41
[17]	<0.7	>17	3.4-10.7		60,>50	NA	0.48×0.12
[18]	<1.95	>6	3.49-11.04		>30,>40	NA	1.04×0.38
[19]	<0.55	>10	3.1-10.0		>55,>40	<0.23	0.5×0.28
[20]	<1.4	>11	3.1-11.1		>40,>20	NA	0.51×0.37
**UWB filter with notch bands implementation**							
[21]	<1	>15	2.7-12.1	5.1,6,8/>15	18,32	NA	0.07×0.02
[22]	0.52	>12	3.25-10.73	5.6,6.42,8.03/>19	>50,>30	NA	1.04×0.66
[23]	<0.9	>12	3.1–10.7	6.5/> 35	>40,>25	<0.4	0.096×0.08
[24]	<1.13	>10	2.73–11.78	5.9/46.3	>40/>40	<0.6	0.32×0.29
[25]	<1.2	>12	2.5-10.96	5.1, 6.45,7.9/>18	>50,>30	<0.45	0.95×0.78
[8]	0.63	10.8	2.9–10.75	5.13, 6.15, 8/ > 18	>40,>30	NA	0.95×0.78
[26]	<15	>15	3.1-10.6	5.18,5.86,7.92/>18	>35,>35	NA	0.71×0.34
[27]	<1.84	>10	2.9-10.3	3.3/>14	>30,>40	<0.5	0.35×0.79
[2]	<1.74	>12.15	3.76-11.29	9.28,10.48/>20	>45,>30	<1.5	0.20×0.2
this	0.61	>18	3.52-11.68	6.80,9.82/>26	>47,>54	<0.24	0.41×0.20

Ref.:Reference;NB(GHz):notch band;λ_*g*_:the guided wavelength at 7.59GHz.

## 5 Conclusion

This manuscript introduces an optimized evolution of the conventional half-wavelength SIR T-type configuration, leading to the development of a novel stepped-triangular resonator. The resonator is tightly coupled with both the input and output feed lines, thereby forming a UWB structure. The 3 dB bandwidth spans a wide frequency range from 3.52 to 11.68 GHz, centered at 7.59 GHz. By employing asymmetric branches and coupling SRR techniques, two notches are generated at 6.80 GHz and 9.82 GHz, respectively. Lastly, the introduction of a new transmission zero on the right side via the defect ground structure elevates the attenuation from 32.29 dB to 54.52 dB, demonstrating the ability to suppress parasitic passbands. The suppression capability surpasses 30 dB within the 13.41-16.0 GHz range, consequently broadening the attenuation stopband. Moreover, the filter exhibits three transmission zeros, indicating outstanding out-of-band suppression performance. Simulation and physical experimentation conducted using HFSS 15.0 reveal that the UWB filter exhibits favorable passband characteristics and significant attenuation at notch frequencies and effectively introduces sharp roll-offs at the passband edges to mitigate parasitic signals. Additionally, the compact design and minimal footprint of the filter fulfill the criteria for filter miniaturization.
